# Toscana Virus in Wild-Caught Sand Flies in Portugal, Findings from the National Vector Surveillance Network, 2023

**DOI:** 10.3390/pathogens13100905

**Published:** 2024-10-15

**Authors:** Fátima Amaro, Líbia Zé-Zé, Hugo Costa Osório, Patrícia Soares, Manuel Silva, Inês Campos Freitas, Maria João Alves

**Affiliations:** 1 Centre for Vectors and Infectious Diseases Research, National Institute of Health Doutor Ricardo Jorge, Avenida da Liberdade n.º 5, 2965-575 Águas de Moura, Portugal; libia.zeze@insa.min-saude.pt (L.Z.-Z.); hugo.osorio@insa.min-saude.pt (H.C.O.); patricia.soares@insa.min-saude.pt (P.S.); manuel.silva@insa.min-saude.pt (M.S.); ines.freitas@insa.min-saude.pt (I.C.F.); m.joao.alves@insa.min-saude.pt (M.J.A.); 2 Environment and Infectious Diseases Research Group, Environmental Health Institute (ISAMB), 1649-028 Lisboa, Portugal; 3 Center for the Study of Animal Science (CECA), Institute for Agricultural and Agro-Alimentary Science and Technology (ICETA), University of Porto, Praça Coronel Pacheco n.º 15, 4050-053 Porto, Portugal

**Keywords:** phleboviruses, REVIVE network, sand flies, Toscana virus

## Abstract

Phlebotomine sand flies play a crucial role in both human and veterinary medicine, acting as vectors for *Leishmania* parasites and most known phleboviruses. In Portugal, the REVIVE program, a comprehensive national surveillance network under the Ministry of Health, has included sand fly surveys since 2016. REVIVE aims to identify existing sand fly species in the country, determine which pathogens are circulating among them, and provide actionable insights for prevention and control measures when necessary. In this way, annually, from May to October, health technicians collect sand flies across mainland Portugal with CDC light traps. The collected sand flies are sent to the Centre for Vectors and Infectious Diseases Research for species identification and molecular screening of pathogens. On 21 September 2023, Toscana virus (TOSV), a well-known phlebovirus in the Mediterranean region due to its capacity to cause neurological disease, was detected in a pool of 30 sand flies collected in Algarve, the southernmost region of Portugal. A 668 bp partial sequence of the nucleoprotein gene shows similarity with TOSV strains from Spain. To our knowledge, this is the first detection of TOSV in its vector in this country, having previously only been reported in vertebrate hosts. These findings highlight the important role of ongoing surveillance efforts in monitoring and understanding the dynamics of sand fly-borne diseases in Portugal.

## 1. Introduction

Phlebotomine sand flies (family Psychodidae) are small dipterans, with a limited flight range, yet they have a significant impact on human and veterinary health due to their ability to transmit various pathogens. Among these, the most notable concerning human disease are the protozoan parasites of the genus *Leishmania* (Kinetoplastida: Trypanosomatidae), which affect thousands of people worldwide each year. Additionally, sand flies can transmit viruses within the *Phlebovirus* genus (Bunyavirales: *Phenuiviridae*). This is the most important one in terms of Public Health, especially in the countries surrounding the Mediterranean Basin.

Toscana virus (*Phlebovirus toscanaense*, TOSV) was first isolated from *Phlebotomus perniciosus* sand flies during an entomological survey in Tuscany, Italy, in 1971. In the 1980s, the virus was also found in the same region, in *Ph. perfiliewi* specimens [1]. Phlebotomine sand flies are the confirmed vectors of TOSV, which has also been detected in *Sergentomyia minuta* in France, *Ph. sergenti* and *Ph. longicuspis* in Morocco, *Ph. neglectus* in Croatia, and *Ph. tobbi* in Cyprus [1,2,3,4,5,6].

Like other phleboviruses, TOSV has a tri-segmented genome, comprising large (L), medium (M), and small (S) segments that encode the RNA-dependent RNA polymerase, the envelope glycoproteins, and the nucleoprotein, respectively [7]. These genetic characteristics make phleboviruses prone to reassortment and recombination events [8]. The significance of TOSV as a human pathogen became evident in 1983 when it was isolated from the cerebrospinal fluid of a young woman hospitalized with aseptic meningitis in Tuscany [9]. That same year, a Swedish tourist fell ill after vacationing in Albufeira, south of Portugal, making this the second country recognized as endemic for TOSV [10]. Since then, TOSV has been identified as a cause of disease in European countries bordering the Mediterranean, including most Mediterranean islands, the Balkan peninsula, and also North Africa [11]. While TOSV can cause asymptomatic or mild flu-like infections, its neurotropic nature and potential to cause aseptic meningitis or meningoencephalitis makes it the most significant sand fly-borne phlebovirus in terms of Public Health. Although most TOSV infections are benign and self-limiting, six fatalities have been reported: one in Italy and five in Romania [12,13].

The natural cycle of TOSV still remains unclear, as its vertebrate reservoir has yet to be identified. The brief duration of viremia and the absence of persistent infection suggest that humans do not play a role in the natural maintenance of TOSV [14]. In the search for a non-human vertebrate reservoir, the first isolation of TOSV was achieved in Italy, in a brain sample from a bat (*Pipistrellus kuhlii*) in 1988 [1]. Given that this isolation occurred simultaneously with others from sand flies, and no subsequent reports have confirmed the presence of the virus in bats—along with initial tests showing no hemagglutination-inhibiting antibodies in the bat—it has been suggested that this TOSV isolation may have been the result of cross-contamination [15].

Over the years, antibodies against TOSV have been reported in dogs, cats, and livestock in Mediterranean countries [16,17,18,19,20,21,22,23,24,25]. Birds are gaining more attention as potential reservoirs due to their capacity to fly over great distances. Supporting this hypothesis, high rates of antibodies against TOSV were found in common quails (*Coturnix coturnix*) in Spain [26]. Additionally, in Turkey, TOSV sequences were identified in organ samples from a greater flamingo (*Phoenicopterus roseus*), a great white pelican (*Pelecanus onocrotalus*), and a black stork (*Ciconia nigra*), though virus isolation was unsuccessful [27]. Subsequent research in organ samples originating from birds collected in the same country led to virus isolation from pigeons (*Columba livia*), mallards (*Anas platyrhynchos*), and partridges (*Perdix perdix*), marking the first isolations of this phlebovirus in avian species and suggesting that migratory birds could be responsible for its spread between countries [28].

In Portugal, following the initial reports of tourists who fell ill after vacationing in Albufeira (1983) and Coimbra (1996) [10,29], TOSV has been confirmed in humans, cats, and dogs through several seroepidemiological and retrospective studies [30,31,32]. In response to diagnostic requests to the National Reference Laboratory, between 2009 and 2018, five human cases of TOSV were confirmed among 608 patients presenting with compatible symptoms. Since then, no further TOSV infections have been reported and the virus has never been detected in entomological surveys [33,34].

Since 2008, the REVIVE program, a comprehensive national surveillance network working under the Ministry of Health has been operational, initially targeting mosquitoes and later expanding to include ticks in 2011 and sand flies in 2016. The primary objectives of REVIVE concerning sand flies are to identify the existing species, ascertain which pathogens are circulating among them, and provide actionable insights for prevention and control measures if needed. This work aims to report the first detection of TOSV in wild-caught phlebotomine sand flies in Portugal during the REVIVE activities in 2023.

## 2. Materials and Methods

### 2.1. Sand Fly Collections

As part of the REVIVE, health technicians conduct annual sand fly collections across mainland Portugal from May to October, using miniature CDC light traps equipped with adapted collection bags (John W. Hock Company, Gainesville, FL, USA) and baited with dry ice whenever possible. In 2023, following established network protocols, the traps were set from sunset to sunrise in nearby animal facilities or in rural settings across the country, operating for one or two nights each month. These collections, specifically focused on sand flies, will henceforth be referred to as “targeted collections”. Additionally, while health technicians conducted mosquito trapping sessions using CDC light traps and BG-Sentinel traps (Biogents AG, Regensburg, Germany), sand flies were occasionally captured. These “incidental collections” refer to instances where sand flies were found in traps meant for mosquito collection. When this occurred, the sand flies were processed as well.

The collected sand flies from each region were refrigerated and sent to the Centre for Vectors and Infectious Diseases Research at the National Institute of Health.

### 2.2. Sand Fly Identification

In the laboratory, sand flies were sorted by collection site and date, and then stored at −80 °C. Male sand flies were cleared in Marc André’s solution, mounted on spot slides, and examined under a stereomicroscope for species-level identification using established taxonomic keys [35]. For female sand flies, if only a single specimen was available, molecular identification was performed using the cytochrome c oxidase subunit I (COI) gene from mitochondrial DNA, following previously described methods [36].

### 2.3. Viral Detection

Since the primary objective of the surveillance network is to detect vector-borne pathogens and only the female sand flies are blood-feeders, the majority of the male insects were identified and did not proceed for pathogen screening. On the other hand, females were promptly organized into pools of a maximum of 30 sand flies according to site and date of collection. Following nucleic acid extraction (NUCLISENS easyMAG, bioMérieux, Marcy-l’Étoile, France), a pan-phlebo RT-PCR targeting a 370-nucleotide region of the S segment was used to detect phleboviruses’ RNA [37]. Additionally, a generic RT-nested PCR assay using degenerated primers was performed in the positive samples, targeting a 560-nucleotide region of the same segment [38]. Moreover, if the samples tested positive, isolation attempts were made in VERO E6 cells, as previously described [39]. The PCR products were visualized on GelRED (Biotarget, Lisbon, Portugal)-stained 1.5% agarose gel electrophoresis, purified, and sequenced on an ABI 3130xl Genetic Analyzer (Applied Biosystems, Foster City, CA, USA). Homology searches were performed using the BLASTN algorithm (National Center for Biotechnology Information, NCBI), and partial sequences aligned with sequences retrieved from GenBank (National Center for Biotechnology Information, NCBI) via Clustal W in Bioedit version 7.2.5 and further used to build the Maximum Likelihood phylogenetic tree (1000 bootstraps) in MEGA 11 [40,41,42].

## 3. Results

### 3.1. Sand Fly Collections and Identification

**Figure 1:**
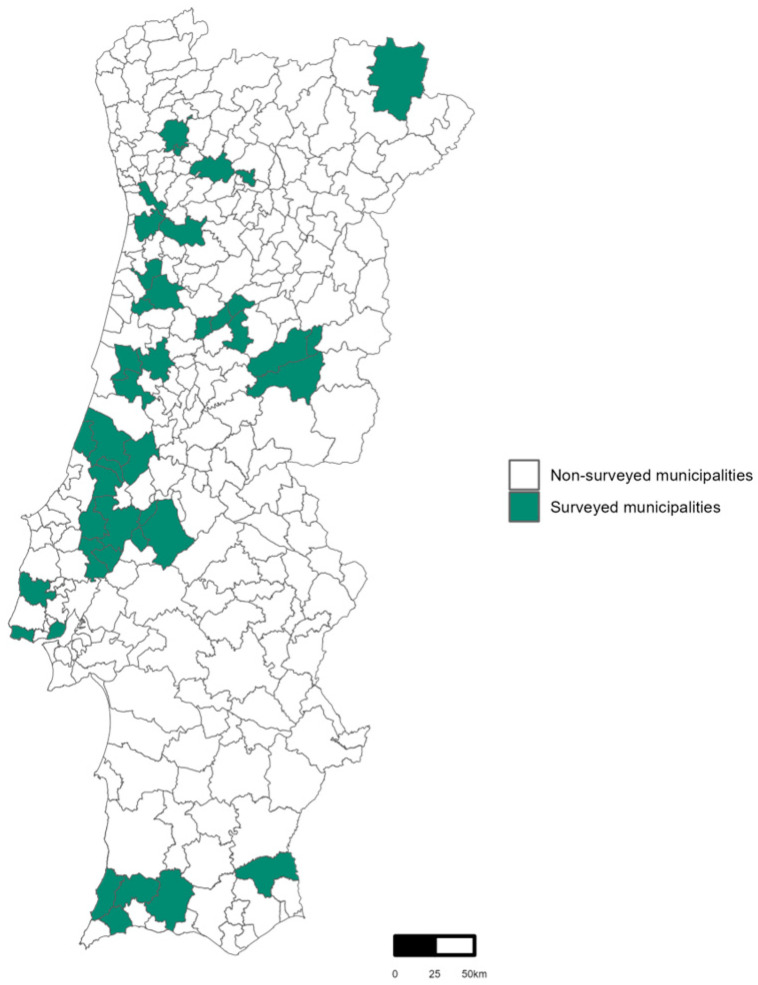
Map of mainland Portugal highlighting all the municipalities where sand flies targeted collections took place in 2023.

In 2023, the Regional Health Administrations of the Algarve, Centre, Lisbon and Tagus Valley, and North carried out 207 trapping sessions (targeted collections), covering 41 municipalities of mainland Portugal (Figure 1). Sand flies were present in 18 of those collections (8.7%), yielding 101 specimens. Additionally, 15 incidental collections were made in 12 municipalities within regions of Algarve, Alentejo, Centre and Lisbon, and Tagus Valley where sand flies were found during mosquito trapping efforts. These incidental collections yielded 660 sand fly specimens.

In 2023, a total of 761 sand flies were collected, consisting of 277 males and 484 females. A total of 130 sand flies (119 males and 11 females) were identified across four species: *Phlebotomus ariasi* (n = 8), *Ph. perniciosus* (n = 118), *Ph. sergenti* (n = 1), and *Sergentomyia minuta* (n = 3) (Table 1).

Notably, a single collection conducted on September 21 in Portimão, located in the Faro district of southern Portugal, yielded a total of 594 sand flies. After consulting with the local health technicians, it was confirmed that the CDC trap that captured the unusually high number of sand flies had been suspended in an ancient olive tree characterized by numerous cavities situated in a rural area. Numerous snail shells and insect remains were observed at the base of the tree. The surrounding environment features diverse vegetation, including scrubland, a pond with a reedbed, a wooded area with tall pine trees, several olive and bay trees, an orchard, and a vegetable garden. The property, located approximately one kilometer from the coast in the Algarve region of southern Portugal, includes an educational center and is an internationally recognized bird-ringing station within one of the largest wetlands in the western Algarve, namely Ria de Alvor [43,44].

**Figure 2:**
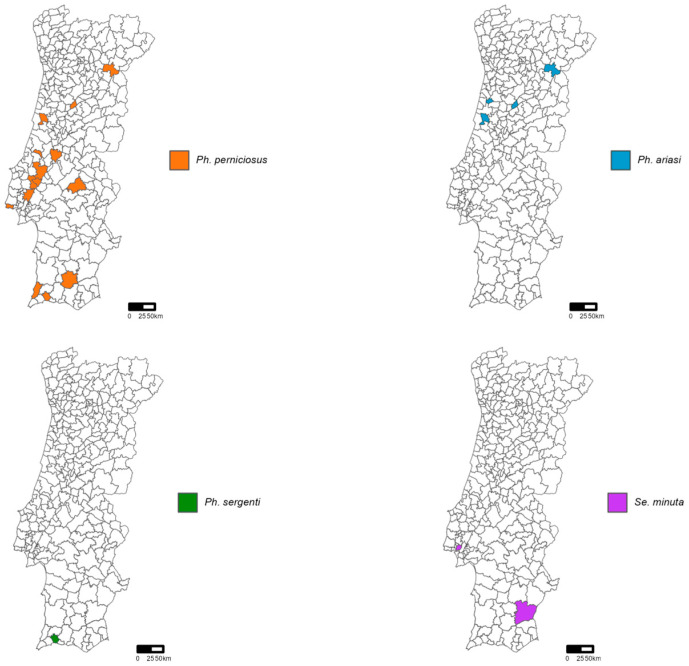
Map of mainland Portugal showing the distribution of the identified specimens, including both from targeted collections and incidental collections.

Figure 2 illustrates the distribution of the identified species by municipality, including both targeted and incidental collections.

### 3.2. Viral Detection

A total of 642 sand flies (155 males and 487 females) organized in 45 pools were screened for the presence of phleboviruses, including 55 from targeted collections and 587 from incidental collections (Table 2).

**Figure 3:**
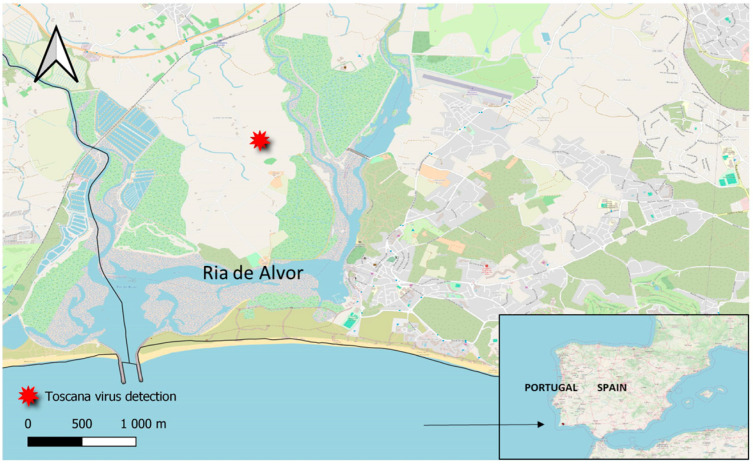
Map showing the location of TOSV detection in Quinta da Rocha near Ria de Alvor.

One pool of 30 female sand flies from the Portimão (Algarve) collection in September tested positive for phlebovirus RNA. The location of the TOSV detection is shown in Figure 3.

**Figure 4:**
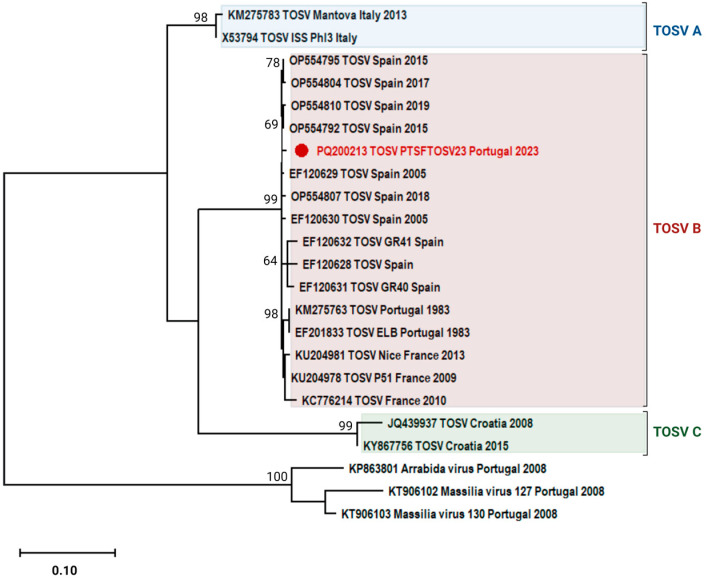
Maximum likelihood phylogenetic tree of phleboviruses’ partial nucleocapsid nucleotide sequences (segment S, 668 positions in the final dataset) using Kimura 2-parameter model and discrete Gamma distribution. Sequences are designated by the GenBank accession number, virus ID, location, and year of detection (whenever available). The sequence marked with a colored red bullet has been sequenced in this study. *Phlebovirus toscanaense* lineages, TOSV A, B, and C are highlighted in blue, red, and green, respectively. Massilia phleboviruses (*Phlebovirus massiliaense*) sequences (KP863801, KT906102, and KT906103) detected in Portugal were used as outgroups. Bootstrap support of over 60% is presented on branches. Composite figure created with Biorender.com.

A genomic sequence of 370 bp of the S segment was retrieved but the isolation attempts were not successful. An additional sequence of the S segment with 668 bp (GenBank accession number PQ200213) was obtained using the generic RT-nested PCR assay. BLASTN homology searches revealed that our sequence belongs to the *Phlebovirus toscanaense* species. The phylogenetic tree based on the partial sequence of the nucleoprotein gene confirmed the clustering of the obtained sequence with the strains circulating in Spain, France, and previously detected in Portugal (first human TOSV infection in 1983) which belong to lineage B (Figure 4).

## 4. Discussion

Among the five species known to occur in Portugal, namely, *Ph. ariasi*, *Ph. papatasi*, *Ph. perniciosus*, *Ph. sergenti*, and *Sergentomyia minuta*, *Ph. perniciosus* was the most frequently identified in 2023, consistent with existing data indicating it as the most widespread sand fly species in the country [45]. Although in much lower numbers, *Ph. ariasi* was the second most frequently identified species in our samples. It is reported to be prevalent in certain regions of Portugal, particularly in the north, which aligns with our findings [35,46].

It was not possible to determine the species of the infected sand flies because the pool contained 30 insects, and at least two species (*Ph. perniciosus* and *Ph. sergenti*) coexist at the collection site. However, it is highly likely that *Ph. perniciosus* is the implicated species, as it is recognized as the primary vector in Spain, our neighboring country [47]. Regrettably, the sample in which TOSV was detected was obtained from an incidental collection and was not processed immediately upon arrival at the laboratory. When a field visit to the collection site was conducted some weeks later, it was not possible to ascertain the cause of the unusually high number of sand flies. As previously described, the trapping site was an ancient olive tree featuring multiple cavities. The presence of invertebrate remains indicated that it may have functioned as a feeding site for vertebrate animals; however, no den or nest associated with a sand fly breeding site was identified during the investigation. The area was densely overgrown with weeds, which hindered thorough exploration.

The detection of TOSV at a site bordering one of the major wetland areas in the Algarve region—part of the Natura 2000 Network and listed under the Ramsar Convention, with recorded observations of over 300 bird species and around 180 species seen annually [48]—revives the discussion about the potential role of avian species as TOSV reservoirs. For instance, mallards, which have been confirmed as carriers of TOSV, can be observed year-round in the region. Additionally, quails and flamingos, which have shown the presence of antibodies or TOSV RNA, are also commonly found throughout the year [48]. Some investigations have indicated that various avian species may serve as viable blood sources for certain sand fly populations, largely due to their relatively passive defense mechanisms compared to other fauna [26]. To date, no birds have been observed exhibiting symptomatic infections of TOSV. However, the occasional transmission of viruses from asymptomatic host species to new hosts has been shown to cause a spectrum of outcomes, from subclinical infections to severe disease or death [49]. Birds possess various physiological and ecological traits related to flight that make them well-adapted to hosting, dispersing, and transmitting viruses, which may also apply in the case of TOSV.

As part of the REVIVE network, TOSV was detected for the first time in sand fly specimens collected in Portugal. The first recorded human cases of TOSV infection in the country occurred less than 45 km from where this current detection was made. Retrospective seroprevalence studies conducted on patients between 2004 and 2008 revealed the presence of antibodies (IgG and/or IgM) in individuals from Algarve, among others [50]. Additionally, request-based diagnostic tests at CEVDI/INSA confirmed acute infections in the same region in 2010 and 2012 [51]. While the incidence of this virus appears to be low—or at least underreported—our findings indicate that it continues to circulate in the area. A more recent serological study published in 2022 surveyed a human population in the Setúbal district, southwest of Portugal, and neutralizing antibodies to TOSV were detected in 5.3% of 400 human sera tested [52]. These findings suggest that the number of TOSV infections in our country may be significantly underestimated. This underestimation could be due to the fact that most TOSV infections go unnoticed because they are asymptomatic or present only mild symptoms. Furthermore, there is a general lack of awareness among physicians regarding the potential of TOSV to cause central nervous system disease. Regardless of the symptoms, TOSV infection is not a notifiable disease at the EU/EEA level and there is no EU case definition. However, in Italy, where the virus was first detected and where it causes a high number of infections annually, TOSV infections are notifiable since 2020 [53,54]. If the same reporting system were in place in Portugal, the virus would likely be more widely recognized.

The sequences from the first reported TOSV infection in Portugal (ELB strain) obtained from the Swedish tourist’s isolate in 1983 were deposited in GenBank, namely, a partial sequence of the polyprotein gene and the complete S segment [55,56]. A retrospective study, conducted in the north region of Portugal included 106 samples from patients admitted to hospitals in the metropolitan area of Porto between 2002 and 2005, presenting with aseptic meningitis; six of these samples tested positive for TOSV RNA using a commercial nested RT-PCR assay [57]. Unfortunately, no genomic sequences were made available from this study. Consequently, apart from the sequences obtained from the original 1983 isolate in different laboratories, the only other genomic sequence of Portuguese origin is a small fragment (111 bp) of the S segment of TOSV obtained from a dog in 2016 [32,58]. In this work, we present not only the first genomic sequence obtained from the sand fly vectors in Portugal but also the first sequence in nearly two decades. The detected strain, PTSTOSV23/INSA2023, belongs to TOSV lineage B and has a higher similarity to strains detected in human infections in Spain since 2005 than to the ELB strain previously detected in Portugal. This result highlights the possible circulation of several TOSV strains.

## 5. Conclusions

In this study, we report the first detection of TOSV in sand flies collected in Portugal, confirming the circulation of this neurovirulent virus at least in the southern region. TOSV is an emerging arbovirus that warrants attention due to its potential to cause human disease. The surveillance conducted by REVIVE provides important insights into the ecology and distribution of sand flies and the pathogens they carry within our country. These findings may be crucial for elaborating prevention and control strategies should outbreaks occur.

## Figures and Tables

**Table 1:** Sand flies collected in 2023 by place, type, and date of collection.

Regional Health Administration	County	Collection Date	Number of Collected Sand Flies (Identified Specimens)
Alentejo	Almodôvar (Inc)	11 October	1 f (1 f *Se. minuta*)
	Avis (Inc)	11 October	1 m (1 m *Ph. perniciosus*)
	Mértola (Inc)	20 September	1 f (1 f *Se. minuta*)
Algarve	Aljezur (Targ)	21 September	1 f (1 f *Ph. ariasi*)
	Portimão (Inc)	25 May	3 m (3 m *Ph. perniciosus*)
	Portimão (Inc)	20 September	1 m + 6 f (1 m *Ph. perniciosus*)
	Portimão (Inc)	21 September	214 m + 380 f (56 m + 3 f *Ph. perniciosus*; 1 f *Ph. sergenti*)
Centre	Batalha (Targ)	24 May	2 m (2 m *Ph. perniciosus*)
	Batalha (Targ)	25 May	2 m + 1 f (2 m *Ph. perniciosus*)
	Carregal do Sal (Targ)	23 May	2 m + 2 f (2 m *Ph. ariasi*)
	Carregal do sal (Targ)	24 May	1 m + 1 f (1 m *Ph. perniciosus*)
	Montemor-o-Velho (Targ)	19 September	3 f (not identified)
	Montemor-o-Velho (Targ)	20 September	3 m + 1 f (1 m *Ph. ariasi*, 2 m *Ph. perniciosus*)
	Oliveira do Bairro (Inc)	25 May	1 m + 2 f (1 m *Ph. ariasi*)
	Pedrógão Grande (Inc)	25 May	1 f (not identified)
	Vila nova de Foz Côa (Inc)	14 June	8 m + 32 f (3 m *Ph. ariasi,* 5 m *Ph. perniciosus*)
Lisbon and Tagus Valley	Azambuja (Targ)	06 August	11 m + 22 f (11 m *Ph. perniciosus*)
	Azambuja (Inc)	30 September	1 m + 2 f (1 m *Ph. perniciosus*)
	Cartaxo (Targ)	05 October	1 m + 3 f (1 m *Ph. perniciosus*)
	Cascais (Targ)	03 May	2 m + 5 f (2 m *Ph. perniciosus*)
	Cascais (Targ)	24 May	1 f (not identified)
	Cascais (Targ)	20 September	2 m + 3 f (2 m *Ph. perniciosus*)
	Cascais (Targ)	27 September	10 m + 1 f (10 m *Ph. perniciosus*)
	Cascais (Targ)	11 October	2 m (2 m *Ph. perniciosus*)
	Cascais (Targ)	18 October	5 m + 2 f (5 m *Ph. perniciosus*)
	Cascais (Targ)	25 October	1 f (not identified)
	Coruche (Inc)	14 September	2 f (2 f *Ph. perniciosus*)
	Lisboa (Inc)	19 September	1 m (1 m *Se. minuta*)
	Santarém (Targ)	25 September	2 m + 7 f (2 m *Ph. perniciosus*)
	Santarém (Targ)	26 September	1 m + 1 f (1 m *Ph. perniciosus*)
	Tomar (Inc)	26 September	1 m (1 m *Ph. perniciosus*)
	Vila Franca de Xira (Inc)	30 May	1 f (1 f *Ph. perniciosus*)
	Vila Franca de Xira (Inc)	03 October	1 f (1 f *Ph. perniciosus*)
**Total**			**277 m + 484 f (119 m + 11 f)**

Targ—targeted collections; Inc—incidental collections; m—male; f—female.

**Table 2:** Sand flies screened for the presence of phleboviruses.

Regional Health Administration	Number of Pools	Number of Screened Sand Flies
Alentejo	2	2 f
Algarve	21	545 (155 m + 390 f)
Centre	8	43 f
Lisbon and Tagus Valley	14	52 f
Total	45	642 (155 m + 487 f)

m—male; f—female.

## Data Availability

Data are contained within the article.
